# Distinct material traits shape soil-dependent N_2_O responses to Fe-, Ca- and Se-modified biochars

**DOI:** 10.3389/fmicb.2026.1972654

**Published:** 2026-09-23

**Authors:** Yuan Diao, Yinan Liu, Hanbing Li, Sumei Li, Xiaowei Wang, Sha Chen

**Affiliations:** 1 College of Environmental Science and Engineering, Key Laboratory of Beijing on Regional Air Pollution Control, Beijing University of Technology, Beijing, China; 2 Environmental Testing and Experiment Center, Chinese Research Academy of Environmental Sciences, Beijing, China

**Keywords:** bacterial community, denitrification genes, metal-modified biochar, nitrous oxide, soil nitrogen cycling

## Abstract

**Introduction:**

Modified biochars are widely proposed as amendments for regulating soil nitrogen cycling, yet the material traits that underpin their contrasting effects on nitrous oxide (N_2_O) emissions remain unclear.

**Methods:**

We compared unmodified biochar with Fe-, Ca-, and Se-modified biochars in loess and black soils during a short-term incubation. Material characterization was combined with N_2_O flux measurements, soil pH and inorganic-N dynamics, bacterial community profiling, and qPCR quantification of nosZ, nirK, and nirS.

**Results:**

XPS, Raman, and FTIR analyses showed clear differences in surface elemental composition, carbon structure, and functional groups among the modified biochars. FeBC consistently reduced cumulative N_2_O emissions in both soils, whereas SeBC increased cumulative N_2_O emissions in loess soil; all biochar treatments reduced N_2_O emissions in black soil. Among the measured chemical variables, NH_4_^+^-N showed the closest association with cumulative N_2_O emissions, while pH and NO_3_^-^-N reflected the broader N-transformation environment. FeBC was associated with bacterial community shifts linked to lower N_2_O emissions, whereas CaBC increased nosZ and nirS abundances in loess soil.

**Discussion:**

The results show that Fe-, Ca-, and Se-modified biochars regulate N_2_O emissions through distinct material-soil-microbial pathways, with their mitigation potential strongly shaped by soil background. These findings provide a trait-based basis for the soil-specific screening of modified biochars for N_2_O mitigation.

## 1 Introduction

Nitrous oxide (N_2_O) emissions from agricultural soils arise from highly dynamic nitrogen transformations (Wang et al., 2026a). Fertilizer input, residue turnover, soil moisture and microbial activity can create neighbouring microsites in which nitrification, nitrifier-denitrification, denitrification and abiotic reactions occur simultaneously or sequentially (Chapuis-Lardy et al., 2006; Sennett et al., 2024; Xiong et al., 2025; Wang et al., 2026b). Net N_2_O emission therefore reflects the balance between production and reduction. This makes mitigation challenging, because an amendment that suppresses one source process may also affect substrate availability, pH, oxygen diffusion, or the microbial capacity for N_2_O reduction (Li et al., 2021; Zhang et al., 2021).

Biochar has been widely studied as a soil amendment for mitigating N_2_O emissions, but its effects vary substantially among biochar materials and soil types (Kaur et al., 2022; Liu et al., 2026). Feedstock, pyrolysis conditions and post-treatment modification can alter pH, ash content, surface chemistry, porosity, nutrient retention and redox reactivity (Davys et al., 2023). Elemental modification further increases this variability by introducing distinct mineral-associated and reactive surface features. For example, Fe-bearing surfaces can introduce mineral and redox-sensitive interfaces (Zhang Y. et al., 2024; Luo et al., 2025), Ca modification may enhance alkalinity and pH buffering (Li et al., 2023; Sun et al., 2023), and Se modification may alter element-specific chemical and microbial responses (Huang et al., 2024; Qin et al., 2024). These differences suggest that modified biochars should be evaluated not only by the introduced element, but also by how their material traits interact with soil chemical and microbial conditions (Zhang et al., 2015; Li et al., 2018).

A central knowledge gap is that modified biochars are often evaluated by treatment label without explicitly linking measured material traits to their soil-specific N_2_O responses. This gap is especially relevant for element-modified biochars, as few studies have directly compared modified biochars across contrasting soils while integrating material traits, inorganic N dynamics, bacterial communities, and denitrification-related genes (Liao et al., 2021; Liao et al., 2026). Because surface elemental composition, carbon structural order, functional groups and pore architecture can influence N retention, gas diffusion, redox conditions and microbial habitat (Wu et al., 2022; Guo et al., 2023), their effects must be interpreted in relation to the physicochemical background of the target soil (Luo et al., 2025; Kalu and Li, 2026). Such an integrated approach can help explain why the same modification mitigates N_2_O emissions in one soil but has neutral or adverse effects in another.

Here, we compared unmodified biochar with Fe-, Ca-, and Se-modified biochars in two contrasting agricultural soils, namely loess soil and black soil. These soils differed markedly in total N, cation-exchange capacity, nutrient status, and texture, providing distinct backgrounds for testing amendment-soil interactions. We hypothesized that (i) FeBC, CaBC, and SeBC would produce distinct rather than interchangeable effects on N_2_O emissions across the two soils; and (ii) these effects would be mediated by soil-specific changes in pH and inorganic-N dynamics, accompanied by treatment-dependent shifts in bacterial communities and denitrification-related genes. To test these hypotheses, we combined multi-technique material characterization with N_2_O flux measurements, soil chemical analyses, bacterial community profiling, and qPCR quantification of denitrification-related genes to determine how distinct material traits and soil background jointly shaped N_2_O responses.

## 2 Materials and methods

### 2.1 Soil collection and experimental design

Soil samples were collected from the surface (0–20 cm) of agricultural fields in Luochuan County, Yan’an City, Shaanxi Province, and Hailun City, Suihua City, Heilongjiang Province, China. After collection, the soils were air-dried, gently ground, and passed through a 2-mm sieve prior to incubation. The initial physicochemical properties of the two soils are summarized in Supplementary Table S1. Based on their textural characteristics, the two soils were classified as loam soil and sandy soil, respectively, and are hereafter referred to as loess soil and black soil throughout this study.

The experiment comprised five treatments in each of the two soils. For each soil and treatment, three independent jars were assigned to repeated gas sampling and three independent jars were assigned to soil sampling, giving 30 incubation units per soil and 60 units in total. Gas and soil measurements therefore used separate jar sets. Soil-sampling jars were repeatedly subsampled on days 0, 1, 2, 3, 5, and 7; the day-7 subsamples were used for microbial analyses. The treatments were urea-amended soil without biochar (CK); CK plus unmodified straw-derived biochar (BC); CK plus Fe-modified biochar (FeBC); CK with Ca-modified biochar (CaBC); CK with Se-modified biochar (SeBC). Each jar contained 500 g dry-weight-equivalent pretreated soil. Biochars were mixed into soil at 2% (w/w), and urea was added at 100 mg N kg^−1^ dry soil (equivalent to 200 kg N ha^−1^). All jars were incubated at 25 °C in darkness for 7 days, and soil moisture was maintained at 60% water-holding capacity by daily weighing and water replacement.

### 2.2 Biochar preparation and characterization

Corn straw was used as the feedstock. The washed corn straw was dried by oven, ground, and passed through a 50-mesh sieve. The unmodified biochar (BC) was produced by oxygen-limited pyrolysis of corn straw powder at 450 °C (10 °C min^−1^ ramp, 2 h holding time), followed by cooling, grinding, and passage through a 200-mesh sieve. FeBC and CaBC were prepared by treating corn-straw powder with FeCl_3_ or CaCl_2_, respectively, before pyrolysis under the same thermal conditions as BC. In contrast, SeBC was prepared by loading Na_2_SeO_3_ onto pre-formed BC. Full preparation steps are provided in Supplementary method.

Biochars were characterized using scanning electron microscopy coupled with energy-dispersive X-ray spectroscopy (SEM-EDS), N_2_ adsorption–desorption analysis with Brunauer–Emmett–Teller (BET), X-ray photoelectron spectroscopy (XPS), Raman spectroscopy, and Fourier-transform infrared spectroscopy (FTIR). SEM-EDS was used to examine surface morphology and elemental composition; BET was used to determine specific surface area, pore volume, and pore-size distribution; XPS was used to characterize surface elemental composition and chemical states, including C1s components; Raman spectroscopy was used to evaluate carbon structural order and defects based on the ID/IG ratio; and FTIR was used to identify surface functional groups. Detailed characterization procedures and instrumental parameters are provided in Supplementary methods.

### 2.3 Measurements of N_2_O and soil chemical properties

Gas and soil samples were collected on days 0, 1, 2, 3, 5, and 7. Before gas sampling, jars were opened for 20 min to equilibrate with ambient air. An initial headspace sample was collected, the jar was then closed for 6 h, and a second headspace sample was collected into a 12 mL Exetainer vial. N_2_O was quantified by gas chromatography (Shimadzu GC-2014, Japan). Fluxes were reported as N_2_O-N (μg N kg^−1^ dry soil h^−1^), and cumulative N_2_O–N emissions were calculated for each gas-sampling jar by trapezoidal integration (Fan et al., 2026). Fresh soil was used for NH_4_^+^–N and NO_3_^−^–N measurements with a continuous-flow analyser, and air-dried soil was used for pH measurement (Fan et al., 2026). The full flux equation and unit definitions are provided in Supplementary method.

### 2.4 16S rRNA gene sequencing and real-time quantitative PCR

At the end of the incubation, soil samples collected 10 cm below the surface were stored at −80 °C. DNA was extracted using the E.Z.N.A. Mag-Bind Soil DNA Kit (Omega Bio-tek, M5635-02, USA), and quantified using a Qubit 4.0 fluorometer (Thermo Fisher Scientific, USA). For bacterial community profiling, the V3-V4 region of the 16S ribosomal RNA (rRNA) gene was amplified. Amplicons were sequenced on an Illumina MiSeq platform. Chimeric reads and singleton operational taxonomic units (OTUs) were removed; sequences were clustered into OTUs at 97% similarity, and representative sequences were taxonomically assigned against the Ribosomal Database Project database. Community analyses used the resulting relative-abundance table.

Quantitative real-time polymerase chain reaction (qPCR) measured gene copy numbers for nirK, nirS, and nosZ. NirK and nirS encode copper- and cytochrome cd1-type nitrite reductases, respectively, while nosZ encodes nitrous oxide reductase. Reactions were run on a LightCycler 480 II (Roche) with three technical replicates, followed by dissociation-curve analysis. Ten-fold serial dilutions were used for standard curves. Copy numbers were expressed as copies mg^−1^ soil and log_10_-transformed. The raw index nosZ/(nirK+nirS) was log_10_ (index+10^−12^), and treatment effects were displayed as differences from CK means, reflecting gene-abundance potential (Liao et al., 2021).

### 2.5 Statistical analysis

The effects of different treatments on N_2_O emissions, soil chemical properties, and functional gene abundances were statistically evaluated. Temporal changes in N_2_O flux were analyzed by considering treatment, incubation day, and their interaction. Cumulative N_2_O emissions, soil chemical properties, and qPCR-derived gene abundances were analyzed using biological replicates as the experimental units. For cross-variable analyses, pH was represented by its temporal mean, whereas NH_4_^+^–N and NO_3_^−^–N were represented by their time-integrated areas under the curve. Pairwise comparisons among treatments were conducted with adjustment for multiple comparisons using the Holm method. Statistical significance was assessed at *p* < 0.05. The relationships between them were examined using linear regression and Spearman’s rank correlation analysis. Mantel tests and envfit analysis were further used to evaluate the relationships between environmental variables and bacterial community composition. All statistical analyses were performed using R 4.6.0.

## 3 Results

### 3.1 Element modification altered biochar surface chemistry

Element modification retained the porous framework of the parent biochar but introduced clear differences in surface loading and pore architecture. SEM-EDS showed a honeycomb-like structure for BC, Fe-rich deposits on FeBC, dense Ca-bearing surface features on CaBC and dispersed Se signals on SeBC; the analysed surface regions contained approximately 8.2 wt% Fe, 5.6 wt% Ca and 1.3 wt% Se, respectively (Supplementary Figure S1). All four materials showed type-IV N_2_ adsorption–desorption behaviour (Bostani et al., 2025), whereas FeBC had a bimodal pore-size distribution, CaBC showed larger pore features, and SeBC had lower pore volume and fewer small pores than BC (Supplementary Figure S2).

XPS, Raman and FTIR analyses further resolved the chemical differences among the materials (Figure 1). The XPS survey spectra confirmed Fe2p, Ca2p and Se3d signals in FeBC, CaBC and SeBC, respectively (Figure 1a), while the high-resolution C1s spectra showed modification-related changes in oxygenated carbon components (Figure 1b). Raman analysis showed that the ID/IG ratio decreased from 1.17 in BC to 0.73, 0.85 and 0.92 in FeBC, CaBC and SeBC, respectively (Figure 1c), indicating modification-dependent changes in carbon structural order (Sui et al., 2021). FTIR spectra showed weaker carbonyl features and stronger metal-oxygen–carbon signatures in FeBC and CaBC, whereas SeBC showed lower oxygenated functional-group intensity (Figure 1d). These results demonstrate that Fe, Ca and Se modification changed both surface elemental chemistry and the carbon matrix, providing distinct material bases for their subsequent soil responses.

**Figure 1:**
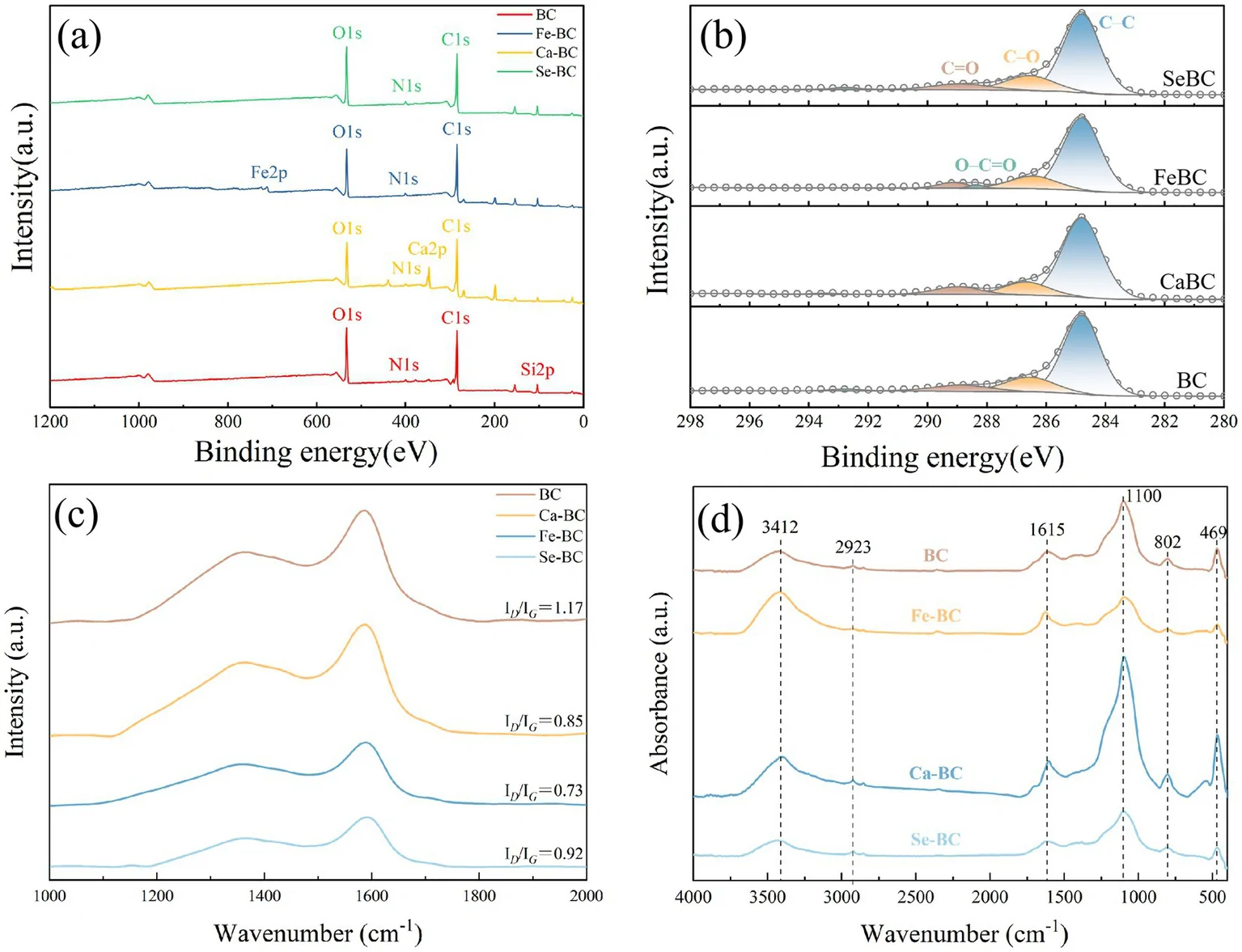
Surface elemental composition and carbon-structure characteristics of unmodified and modified biochars. **(a)** XPS survey spectra, **(b)** high-resolution C1s spectra, **(c)** Raman spectra and ID/IG ratios, and **(d)** FTIR spectra of BC, FeBC, CaBC and SeBC.

### 3.2 Soil-dependent N_2_O responses

Modified biochars produced distinct N_2_O flux patterns during the 7-day incubation, with the largest treatment separation occurring during the early emission pulse. In loess soil, Figure 2a showed a rapid N_2_O increase around day 1 followed by a decline toward day 5–7. FeBC consistently suppressed this early pulse and maintained the lowest flux curve. SeBC retained a higher flux after the peak, especially around day 2. BC remained close to CK, whereas CaBC showed a moderate-to-high early response, demonstrating that the amendments altered emission timing as well as total emission.

**Figure 2:**
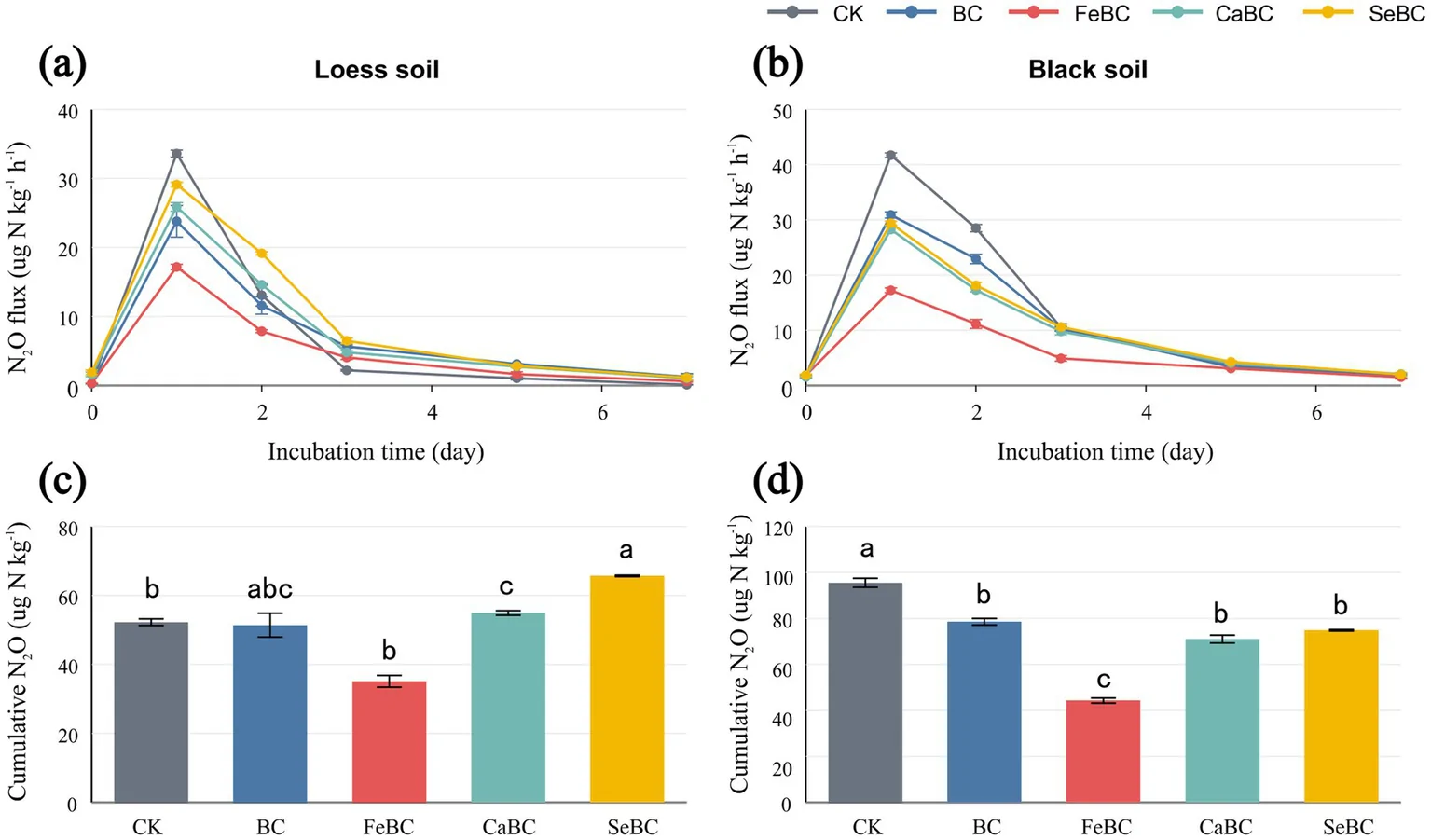
N_2_O emissions following modified biochar application. **(a,b)** N_2_O flux in loess and black soil under CK, BC, FeBC, CaBC, and SeBC at 25 °C and 2% application. **(c,d)** Cumulative N_2_O emissions calculated by trapezoidal integration. Error bars are t-based 95% confidence intervals. Letters denote groups that differed in soil-specific pairwise comparisons after Holm adjustment (*p* < 0.05).

Black soil showed a broader mitigation response to biochar addition. In Figure 2b, CK had the highest early N_2_O pulse, while FeBC formed the lowest curve across the time series. BC, CaBC and SeBC were all below CK after the initial peak. The strongest contrast between the two soils was SeBC: it behaved as a high-emission treatment in loess soil (Figure 2a) but as a mitigating treatment in black soil (Figure 2b). The same Se-modified material produced opposite net responses depending on the receiving soil.

The cumulative-emission panels confirmed the flux trajectories. In loess soil, FeBC reduced cumulative N_2_O–N by 32.8% relative to CK (*p* = 0.0229), whereas SeBC increased it by 25.7% (*p* = 0.0231). In black soil, all biochar treatments were below CK, with FeBC showing the largest reduction (53.6%, *p* = 0.0012), followed by CaBC, SeBC, and BC (Figures 2c,d). Thus, FeBC produced the most consistent reduction across the two soils, whereas the SeBC response depended on soil background.

### 3.3 Inorganic-N trajectories and pH context

Soil pH varied with both soil background and biochar treatment (Figure 3a). Black soil remained near neutral to slightly alkaline throughout the incubation, with relatively small differences among treatments. In loess soil, however, FeBC produced a distinct pH trajectory and remained lower than CK, BC, CaBC, and SeBC for most of the incubation period. This pH separation coincided with the lower N_2_O fluxes and cumulative emissions observed under FeBC (Figures 2a,c), distinguishing FeBC from the CaBC and SeBC treatments.

**Figure 3:**
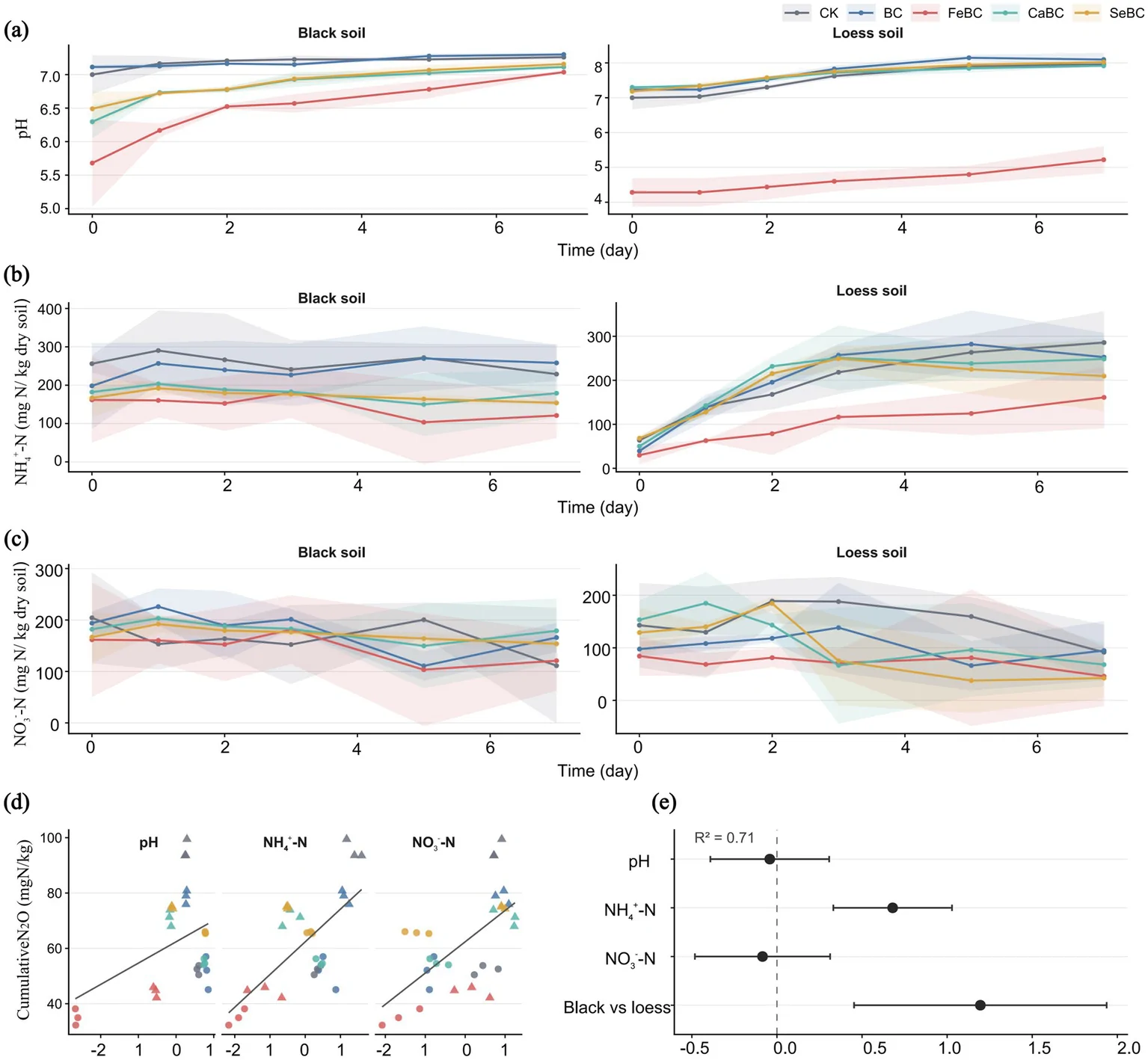
Soil pH and inorganic nitrogen dynamics during incubation. **(a–c)** Temporal changes in pH, NH_4_^+^–N and NO_3_^−^–N, respectively; shaded ribbons are t-based 95% confidence intervals (*n* = 3 independent soil-sampling jars per treatment and day). **(d)** Replicate associations between cumulative N_2_O–N and standardized chemical summaries; pH is the temporal mean, whereas NH_4_^+^–N and NO_3_^−^–N are time-integrated areas under the curve. **(e)** Standardized coefficients and 95% confidence intervals from the multiple linear model relating cumulative N_2_O emission to these chemical summarie and soil background.

NH_4_^+^–N showed the clearest treatment separation among the measured chemical variables (Figure 3b). FeBC maintained the lowest NH_4_^+^–N concentrations in both soils, whereas CK, BC, CaBC, and SeBC showed greater NH_4_^+^–N accumulation, particularly in loess soil. This pattern was consistent with the N_2_O emission results: FeBC combined low NH_4_^+^–N accumulation with low N_2_O fluxes and cumulative emissions, while SeBC maintained higher NH_4_^+^–N in loess soil and produced the highest cumulative N_2_O emissions (Figures 2a,c). Across the core dataset, NH_4_^+^–N showed the strongest positive standardized coefficient for cumulative N_2_O emissions (*β* = 0.680, *p* = 4.75 × 10^−4^), indicating a close association between ammonium availability and N_2_O emission intensity.

NO_3_^−^–N displayed a more dynamic pattern than NH_4_^+^–N, with treatment rankings shifting over time (Figure 3c). FeBC generally had lower or intermediate NO_3_^−^–N concentrations late in the incubation. The association plots and coefficient summary further supported the stronger relationship between NH_4_^+^–N and cumulative N_2_O emissions, whereas NO_3_^−^–N and pH contributed weaker but directionally relevant signals (Figures 3d,e). All of the above results indicate NH_4_^+^–N accumulation, as the main chemical correlate of the treatment-dependent N_2_O responses.

### 3.4 Bacterial community reorganization

Day-7 bacterial community composition was structured by both soil background and biochar treatment. The first two axes of the Bray-Curtis PCoA explained 49.76% of the total community variation, with loess and black soils occupying distinct ordination spaces and treatment centroids shifting within each soil type (Figure 4a). PERMANOVA confirmed significant effects of soil type (*R*^2^ = 0.185, *p* = 0.001) and biochar treatment (*R*^2^ = 0.261, *p* = 0.001), while betadisper analysis provided no evidence that these differences were driven by unequal multivariate dispersion (*p* = 0.774). Environmental fitting further separated cumulative N_2_O emissions and NO_3_^−^–N from pH, NH_4_^+^–N, and the nosZ/(nirK+nirS) potential source-sink index, suggesting that biochar treatments generated distinct microbial-chemical states.

**Figure 4:**
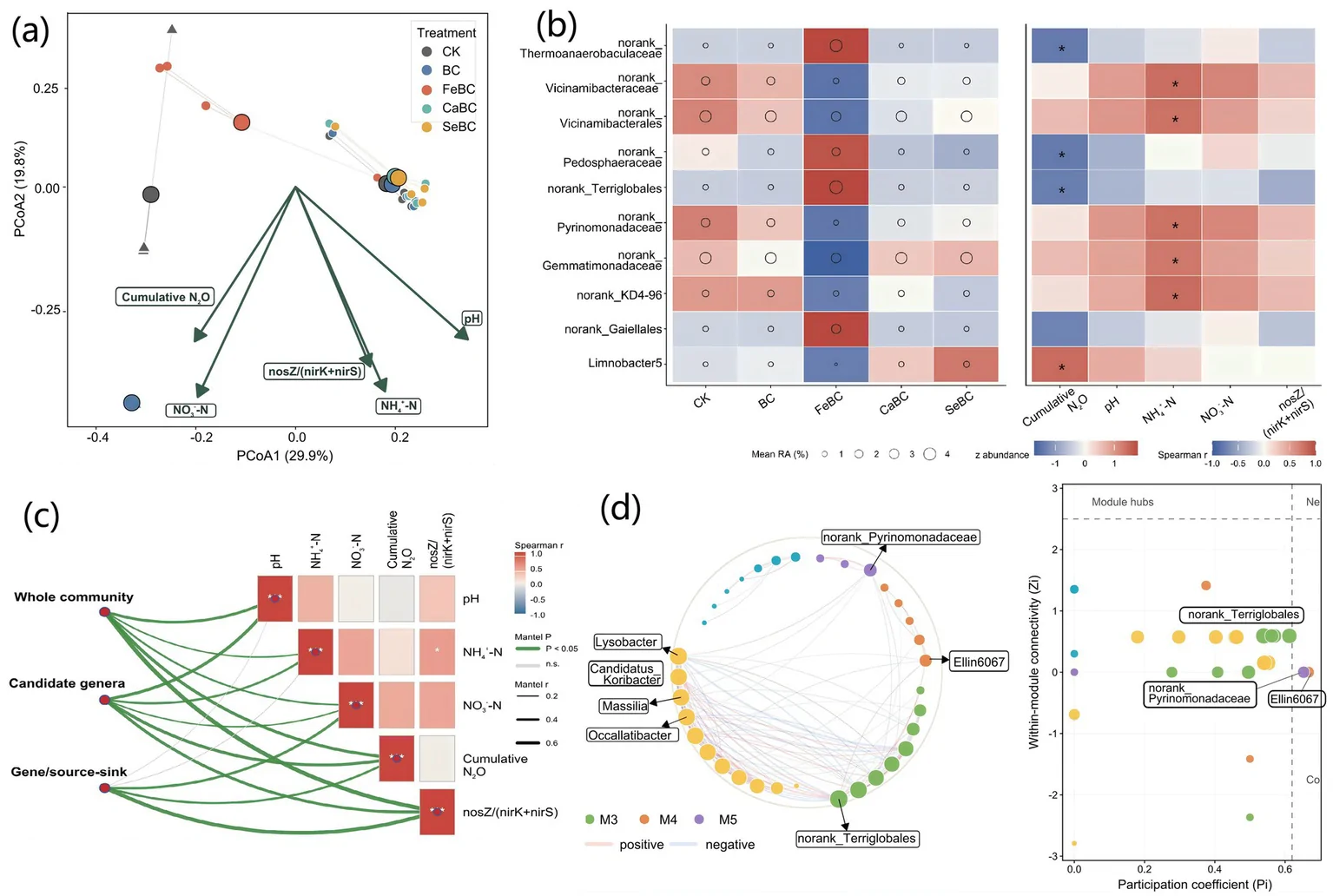
Bacterial community composition and environmental associations. **(a)** Principal coordinate analysis (PCoA) of Bray-Curtis dissimilarities at day 7. **(b)** Loess-soil candidate responsive genera. In the left matrix, background colour is the within-genus *z* score of mean relative abundance and circle area is the treatment mean relative abundance (%); In the right matrix, colour is Spearman’s *r* for associations with each index; *denote BH-adjusted *p* < 0.05. **(c)** Spearman Mantel tests linking whole community, selected-genus, and gene/index distance matrices to measured variables. **(d)** Genus co-occurrence network and Zi–Pi classification; node fill denotes module, node size denotes degree, edge colour denotes correlation sign, and edge width denotes |r|.

In loess soil, FeBC was associated with higher relative abundances of norank_Thermoanaerobaculaceae, norank_Terriglobales, norank_Gaiellales, and norank_Pedosphaeraceae (Figure 4b; Supplementary Figure S5). FeBC had lower relative abundances of Limnobacter and lineages related to Gemmatimonadaceae, KD4-96, Vicinamibacterales, and Pyrinomonadaceae than SeBC or CaBC. Limnobacter correlated positively with cumulative N_2_O–N (*r* = 0.818, BH-adjusted *p* = 0.0179), whereas norank_Terriglobales, norank_Pedosphaeraceae, and norank_Thermoanaerobaculaceae correlated negatively. Gemmatimonadaceae-, KD4-96-, Vicinamibacterales-, and Pyrinomonadaceae-related lineages were positively associated with NH_4_^+^–N. These are treatment and correlation patterns only; they do not assign N_2_O-producing or N_2_O-reducing functions to the taxa (Fan et al., 2026).

Mantel analysis further connected bacterial community structure with soil chemical properties and N_2_O emission patterns. Whole-community Bray–Curtis dissimilarity was significantly associated with pH, NH_4_^+^–N, NO_3_^−^–N, cumulative N_2_O emissions, and the potential source-sink index (Figure 4c). Similar associations were observed for the candidate-genera-based dissimilarity. Network analysis and Zi–Pi classification indicated that these candidate taxa formed distinct ecological modules, with norank_Pyrinomonadaceae and Ellin6067 showing relatively high connectivity within the network (Figure 4d). Together, these results indicate that bacterial community restructuring occurred along the same soil chemical and N_2_O emission gradients that separated FeBC from SeBC and CaBC in loess soil.

### 3.5 Denitrification-gene potential

The response of denitrification-related functional genes to biochar amendments was most pronounced under CaBC and SeBC treatments in loess soil (Figure 5a). CaBC exhibited the highest nosZ abundance, followed by SeBC, whereas FeBC showed a lower nosZ abundance than CK. CaBC also markedly increased nirS abundance, and SeBC showed a comparable enhancement. For nirK, all biochar-amended treatments showed higher abundances than CK, with CaBC and SeBC exhibiting relatively greater increases.

**Figure 5:**
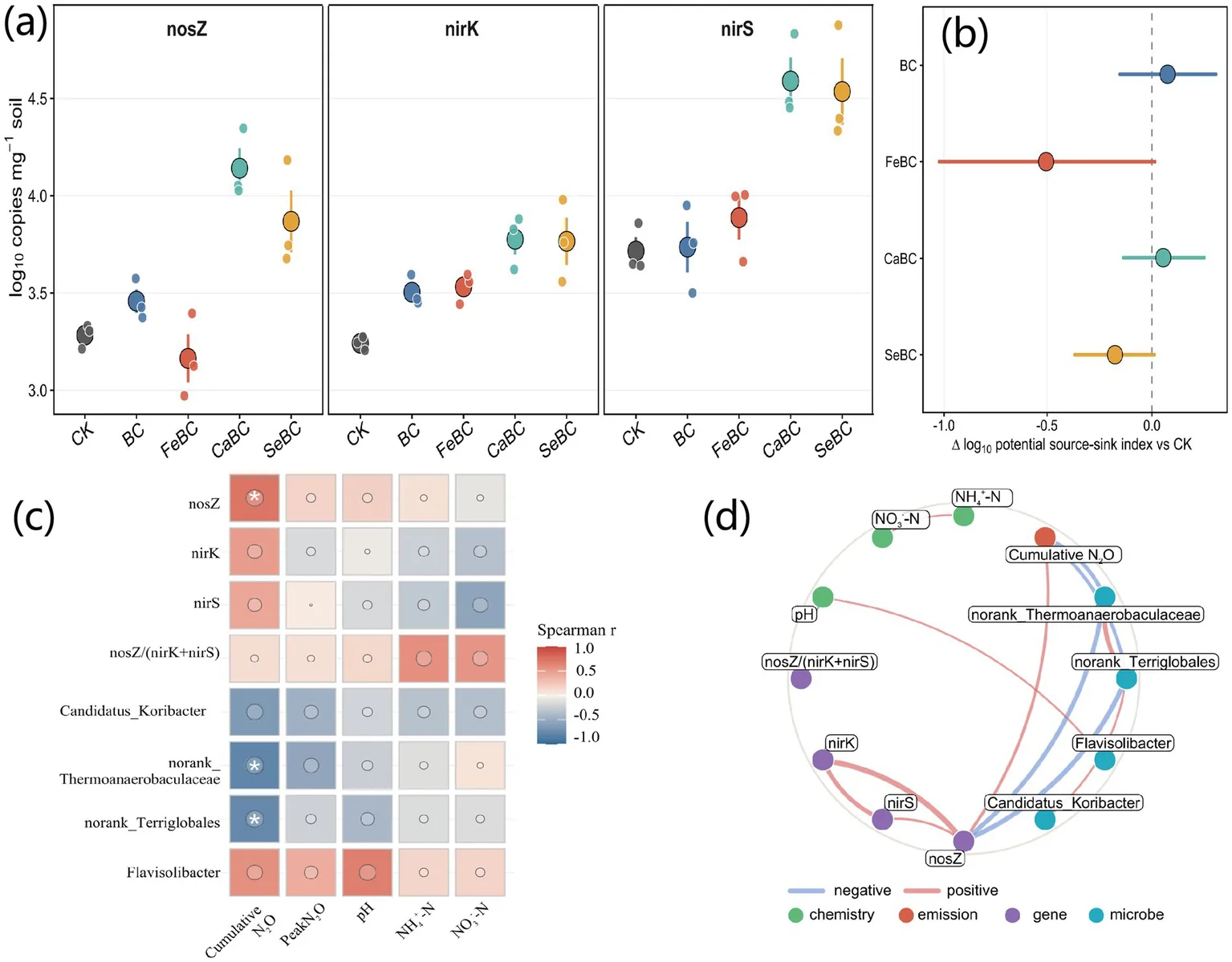
Denitrification-related genes and gene–environment associations. **(a)** NosZ, nirK and nirS copy numbers displayed as log_10_(copies mg^−1^ soil+1); points are biological replicates. **(b)** Treatment effects on the potential source-sink index (log_10_[nosZ/(nirK+nirS) + 10^−12^]), expressed as the difference from the corresponding CK mean. **(c)** Spearman association matrix linking genes, the index, candidate taxa, soil chemistry, and N_2_O–N metrics; tile colour represents r, circle area represents |r|, and *denote BH-adjusted *p* < 0.05. **(d)** Association network; node fill denotes variable type, edge colour denotes association sign, and edge width denotes |r|; edges satisfy |r| > 0.6 and BH-adjusted *p* < 0.05.

The nosZ/(nirK+nirS) ratio provided additional insight into the relationship between denitrification gene abundance and N_2_O emission patterns. As shown in Figure 5b, CaBC exhibited a slightly higher ratio than CK, while BC also showed a positive shift. In contrast, SeBC decreased this ratio, and FeBC exhibited the lowest mean value with relatively large variability. These results suggest that the increase in nosZ abundance under CaBC did not necessarily correspond to a proportional enhancement of the overall N_2_O sink potential, likely because the concurrent increases in nirS and nirK altered the balance between N_2_O production and reduction pathways. Although SeBC promoted nirS abundance, its lower nosZ/(nirK+nirS) ratio was consistent with the higher cumulative N_2_O emissions observed in loess soil (Figure 2c). Notably, FeBC achieved substantial N_2_O mitigation without increasing nosZ abundance or the source-sink index, suggesting that its mitigation effect was more closely associated with changes in inorganic nitrogen availability and microbial community restructuring rather than enhanced genetic potential for N_2_O reduction.

Gene–environment correlations and network analyses revealed distinct microbial associations with N_2_O emissions. In Figure 5c, nosZ abundance was positively correlated with cumulative N_2_O emissions (*r* = 0.736, BH-adjusted *p* = 0.0236). Norank_Thermoanaerobaculaceae and norank_Terriglobales showed negative correlations with cumulative N_2_O emissions, whereas Flavisolibacter was positively associated with both cumulative N_2_O emissions and pH. Network analysis (Figure 5d; Supplementary Figure S7) showed positive associations among nosZ, nirK, and nirS, while cumulative N_2_O emissions were negatively linked with norank_Thermoanaerobaculaceae and norank_Terriglobales. These results highlight the co-variation in functional genes, microbial taxa, and soil properties in regulating N_2_O emission patterns.

### 3.6 Integrated response pattern

The integrated framework summarized the relative contributions of soil properties, microbial community structure, and functional genes to cumulative N_2_O emissions. In Figure 6a, NH_4_^+^-N showed the strongest association with cumulative N_2_O emissions, followed by soil background and NO_3_^−^–N, whereas microbial community axes, nosZ abundance, and the nosZ/(nirK+nirS) index showed relatively weaker associations. The block summary (Figure 6b) further indicated that soil chemical properties provided the primary explanatory layer, while bacterial communities and functional genes contributed additional ecological information. The pathway framework in Figure 6c integrated these relationships into a sequential response model, in which soil background and biochar properties influenced pH and inorganic-N availability, followed by shifts in bacterial communities, functional genes, and ultimately N_2_O emissions. The proposed Fe–N-electron transfer and pore/O_2_/redox pathways represent potential mechanisms underlying the distinct effects of FeBC, CaBC, and SeBC (Oh et al., 2016; Xu et al., 2025; Wang et al., 2026a). Collectively, these results support a soil-amendment interaction framework where biochar-derived properties regulate N_2_O emissions through coupled chemical, microbial, and functional processes.

**Figure 6:**
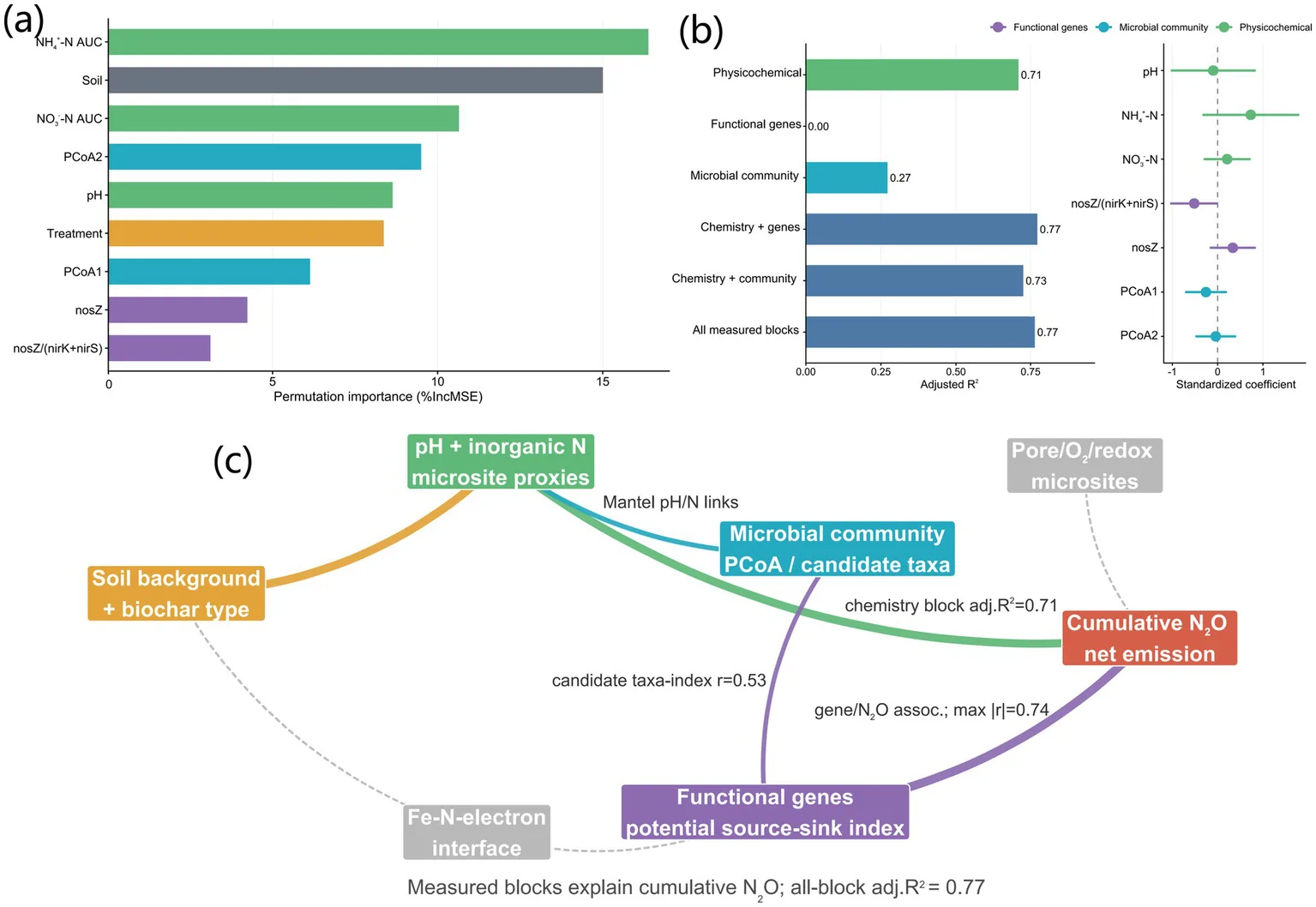
Integrated factors associated with cumulative N_2_O emissions. **(a)** Random-forest permutation importance from 21 complete observations, nine predictors, and 1,000 trees; the final out-of-bag *R*^2^ was 0.716 and importance is the increase in out-of-bag mean squared error after predictor permutation. **(b)** Adjusted *R*^2^ values from least-squares models; coefficient intervals are 95% confidence intervals. **(c)** Integrated association framework combining the chemistry-block adjusted *R*^2^ (0.711), Spearman-Mantel associations from Figure 4, and gene–environment Spearman correlations from Figure 5. Thick solid lines denote the strongest measured associations, thin solid lines denote other measured associations, and grey dashed lines denote literature-supported hypotheses.

## 4 Discussion

### 4.1 Divergent responses of FeBC, CaBC and SeBC

The contrasting N_2_O responses to FeBC, CaBC, and SeBC indicate that elemental modification alone was insufficient to determine the mitigation potential of biochar. Although all three materials were derived from the same straw biochar, they differed in surface elemental composition, carbon structure, functional groups, mineral deposition, and pore architecture (Figure 1; Supplementary Figures S1, S2). These differences provided distinct physicochemical interfaces for soil N transformation, but their effects were expressed differently depending on soil background (Yuan et al., 2025).

Among the three modified biochars, FeBC showed the most consistent mitigation effect. Its Fe-rich surface deposits, Fe 2p signal, bimodal pore-size distribution, enhanced metal-oxygen–carbon features, and lower ID/IG ratio suggest a more reactive mineral-associated surface (Figure 1; Supplementary Figures S1, S2) (Oh et al., 2016; Su et al., 2025). This material profile coincided with suppression of the early N_2_O pulse and the lowest cumulative N_2_O emissions in both soils (Figures 2a–d), with lower NH_4_^+^–N and a distinct pH response (Figures 3a,b). In loess soil, FeBC was also associated with enrichment of Terriglobales- and Thermoanaerobaculaceae-related taxa and a reduction in several NH_4_^+^–N-associated taxa (Figure 4b) (Jiang et al., 2024). However, FeBC did not increase nosZ abundance or the potential source-sink index (Figures 5a,b). Its mitigation effect was more likely related to changes in inorganic N availability and microbial habitat conditions than to enhanced gene potential for N_2_O reduction (Fungo et al., 2019; Khan et al., 2023).

The CaBC response was less straightforward. Its Ca-associated surface features, oxygen-containing functional groups, and pore characteristics suggest potential effects on pH buffering, cation exchange, and N retention (Sun et al., 2023; Hu et al., 2026). These traits were sufficient to reduce cumulative N_2_O emissions in black soil, but in loess soil CaBC had little effect or slightly increased emissions relative to CK (Figures 2c,d). The clearest response in loess soil occurred at the functional-gene level, where CaBC produced the highest nosZ abundance and strongly increased nirS, with nirK also increasing (Figure 5a). Because the potential source-sink index changed only modestly (Figure 5b), the increase in nosZ did not represent a selective strengthening of the N_2_O sink (Meng et al., 2025). Instead, CaBC appeared to broaden denitrification-related gene potential on both the upstream nitrite-reduction and downstream N_2_O-reduction sides, leaving the final emission response dependent on pH and inorganic N availability (Ouerghi et al., 2023).

SeBC further emphasized the importance of soil context. SeBC retained a porous framework relatively similar to BC, with lower pore volume and weaker oxygenated functional group intensity than the other modified biochars (Lai et al., 2025). In loess soil, SeBC maintained higher NH_4_^+^–N, enriched Limnobacter- and Gemmatimonadaceae-related taxa, increased nirS abundance, and lowered the potential source-sink index (Figures 3b, 4b, 5a,b). These responses matched the sustained post-peak N_2_O flux and the highest cumulative N_2_O emissions among the treatments (Figures 2a,c). The same material reduced cumulative N_2_O emissions in black soil, however, indicating that Se modification did not exert a fixed effect on N_2_O production (Figure 2d) (Zhan et al., 2021; Qu et al., 2026). Its performance was likely shaped by differences in nutrient status, exchange capacity, and microbial community background between the two soils (Hou et al., 2025).

### 4.2 Soil background shaped amendment performance

The two soils provided distinct chemical and physical backgrounds for the same set of biochar amendments. Black soil had substantially higher total N (0.922% vs. 0.11%) and CEC (56.8 vs. 12.4 cmol^+^ kg^−1^), as well as higher available K and a coarser particle-size distribution, whereas loess soil had higher available P but lower total N and exchange capacity (Supplementary Table S1).

These differences were reflected in the N_2_O responses. Black soil showed the highest N_2_O emissions under CK, and all biochar treatments reduced cumulative N_2_O emissions (Figures 2b,d). In contrast, loess soil exhibited a much greater divergence among amendments. FeBC consistently reduced NH_4_^+^–N and cumulative N_2_O emissions, whereas SeBC maintained higher NH_4_^+^–N and increased cumulative N_2_O emissions (Figures 3a–c). The microbial responses showed a similar separation, with FeBC favoring taxa associated with lower N_2_O emissions, while SeBC and CaBC were associated with taxa linked to higher cumulative N_2_O emissions or NH_4_^+^–N (Figure 4b). These soil-specific patterns are consistent with the broader conclusion that biochar responses depend on soil background (Wu et al., 2021; Zhang et al., 2026), with the lower-exchange-capacity loess soil showing greater differentiation among material types.

### 4.3 Nitrogen substrates as the chemical link

NH_4_^+^–N emerged as the strongest chemical correlate of cumulative N_2_O emissions, consistent with its central position in soil N turnover and its role as a substrate for subsequent N transformations (Fungo et al., 2019; Li et al., 2021). The contrasting NH_4_^+^–N trajectories were closely aligned with the N_2_O responses of the biochars: FeBC maintained the lowest NH_4_^+^–N concentrations and was associated with lower N_2_O emissions, whereas SeBC maintained higher NH_4_^+^–N and showed greater cumulative N_2_O emissions in loess soil (Figures 2, 3). These patterns suggest that the availability and persistence of ammonium were closely related to the magnitude of N_2_O production under the different amendments (Wen et al., 2026).

NO_3_^−^–N also contributed to the N_2_O response but showed a more dynamic pattern. Its concentration changed over the incubation period and did not maintain a consistent treatment ranking (Figure 3c), reflecting the balance between its production and consumption during N transformation (Zhang et al., 2015; Craswell et al., 2021). Nevertheless, NO_3_^−^–N remained an important variable in the integrated analysis (Figures 6a,c), indicating that nitrate availability contributed to the conditions governing N_2_O production.

Soil pH provided an additional context for these N responses but did not show a uniform relationship with N_2_O emissions (Ouerghi et al., 2023). Notably, FeBC was associated with relatively low pH despite producing the lowest N_2_O emissions (Figures 2c, 3a), indicating that its mitigation effect was unlikely to be explained by pH elevation alone. CaBC, in contrast, showed characteristics consistent with stronger pH buffering and increased denitrification-gene abundance, particularly nosZ and nirS (Figure 5a), but its final emission response depended on substrate availability (Hu et al., 2026).

### 4.4 Microbial indicators and gene potential

Bacterial community responses provided further evidence that distinct microbial states associated with the contrasting N_2_O responses to biochar amendments. In loess soil, FeBC was associated with lower NH_4_^+^–N and N_2_O emissions and a distinct bacterial community enriched in Acidobacteriota-and Actinobacteriota-related lineages, including Terriglobales, Gaiellales, and related taxa (Figure 4b). Gaiellales and related Actinobacteriota lineages can be interpreted as indicators of mineral-soil and organic-matter transformation contexts (Wei et al., 2024). Their distribution was consistent with changes in soil nutrient status and pH, supporting a link between FeBC-induced shifts in the soil environment and bacterial community structure (Yao et al., 2020; Gonçalves et al., 2024; Zhang H. et al., 2024).

By contrast, SeBC and CaBC were linked to a different microbial pattern under the higher NH_4_^+^–N and higher N_2_O conditions in loess soil (Martins et al., 2024; Wang S. et al., 2025). Limnobacter was enriched under SeBC and showed a strong positive association with cumulative N_2_O emissions (Figure 4b), suggesting that it may serve as a substrate- or redox-responsive indicator rather than a specific denitrification marker (Lyu et al., 2025). Gemmatimonadaceae-, Vicinamibacterales-, and Pyrinomonadaceae-related taxa were more closely aligned with elevated NH_4_^+^–N, while Flavisolibacter was positively associated with both cumulative N_2_O emissions (Ramírez et al., 2020) and pH (Figure 5c). These patterns indicate that the contrasting N_2_O responses were accompanied by broader community reorganization (Ali et al., 2022; Gu et al., 2023).

The qPCR results added a functional perspective to this community pattern. In loess soil, CaBC increased both nosZ and nirS abundance, indicating that Ca modification enhanced the genetic potential for both N_2_O reduction and upstream nitrite reduction (Figure 5a) (Zhou et al., 2025). This helps explain why higher nosZ abundance did not necessarily correspond to lower cumulative N_2_O emissions: the final emission response depended on the balance among inorganic N availability, bacterial community structure, and multiple denitrification-related gene pools (Meng et al., 2025; Wang X. et al., 2025). In summary, FeBC mitigation was more closely associated with changes in NH_4_^+^–N, pH, and bacterial habitat structure, whereas CaBC mainly strengthened denitrification-gene potential and SeBC was associated with a higher N-substrate, higher emission microbial state in loess soil.

### 4.5 Implications for biochar design

These findings support a trait-based, soil-specific strategy for modified biochar selection. FeBC showed the most consistent mitigation potential across both soils, suggesting that Fe-bearing surface properties, pore structure, and carbon characteristics can jointly regulate inorganic N availability and microbial habitat conditions (Su et al., 2025; Wang et al., 2026a). In contrast, CaBC mainly altered denitrification-related gene potential and may be more suitable for soils where pH regulation or incomplete denitrification constrains N_2_O reduction (Hu et al., 2026). SeBC showed stronger soil dependence, indicating that the introduction of a functional element does not necessarily improve mitigation unless it matches the N-cycling context of the target soil (Qu et al., 2026).

More broadly, this study highlights the importance of connecting biochar physicochemical traits with soil-dependent N_2_O outcomes across chemical, microbial and gene-level evidence. Fe-, Ca-, and Se-based modifications altered surface reactivity, pH regulation, pore structure, and microbial habitats through different pathways, but their effects on N_2_O emissions were ultimately shaped by soil background. Future screening of modified biochars should therefore integrate material characterization with key target soil properties, including pH, CEC, mineral-N dynamics, and microbial community structure (Kerner et al., 2023; Zhang et al., 2026).

Future studies should incorporate direct measurements of NO_2_^−^–N dynamics, microsite CO_2_ and redox potential (Eh), Fe(II)/Fe(III) speciation, electron-exchange capacity, Se availability, and time-resolved gene expression or enzyme activity. These measurements would help distinguish the relative contributions of nitrification, denitrification, and abiotic reactions to N_2_O production and reduction, and would provide direct tests of the proposed Fe–N-electron transfer and pore/O_2_/redox pathways shown in Figure 6c. Longer incubation experiments and field trials across soils differing in CEC, pH buffering capacity, and mineral-N status would further test the robustness of the amendment-soil interaction framework and guide the practical design of modified biochars for N_2_O mitigation.

## 5 Conclusion

Fe-, Ca-, and Se-modified biochars produced contrasting N_2_O responses in loess and black soils. FeBC consistently reduced cumulative N_2_O emissions across both soils, whereas SeBC increased emissions in loess soil, indicating that elemental modification alone does not guarantee N_2_O mitigation. CaBC mainly altered denitrification-related gene abundance, but did not produce a consistently stronger mitigation effect. Across treatments, NH_4_^+^–N dynamics showed the closest association with cumulative N_2_O emissions, while bacterial community structure and denitrification-gene abundance shifted with changes in pH and inorganic N availability. These results indicate that distinct material traits influenced N_2_O emissions through their interactions with soil chemical conditions and microbial responses. Collectively, this study highlights the need for soil-specific evaluation of modified biochars by integrating material characterization, time-resolved soil chemistry, and microbial indicators, thereby establishing a trait-based framework for N_2_O mitigation screening. This framework can help identify modified biochars with more consistent mitigation potential and avoid amendments that increase N_2_O emission risk under specific soil conditions.

## Data Availability

The datasets presented in this study can be found in online repositories. The names of the repository/repositories and accession number(s) can be found below: https://figshare.com/, 10.6084/m9.figshare.33284988.
