# Supplementary material for: Distinct material traits shape soil-dependent N2O responses to Fe-, Ca- and Se-modified biochars
Source: Front Microbiol. 2026 Sep 23;17:1972654. doi: 10.3389/fmicb.2026.1972654 (PMC13642713; doi:10.3389/fmicb.2026.1972654)
Supplement: Supplementary file 1 [file Data_Sheet_1.docx]

**Supporting Information**

**Distinct material traits shape soil-dependent N_2_O responses to Fe-, Ca- and Se-modified biochars**

Yuan Diao^a^, Yinan Liu^a^, Hanbing Li^a^, Sumei Li^a^, Xiaowei Wang^a, b^, Sha Chen^a, *^

a College of Environmental Science and Engineering, Key Laboratory of Beijing on Regional Air Pollution Control, Beijing University of Technology, Beijing 100124, China

b Environmental Testing and Experiment Center, Chinese Research Academy of Environmental Sciences, Beijing 100012, China,

**Supplementary Methods**

**S1. Biochar preparation and modification**

The parent biochar (BC) was prepared from corn straw. Straw powder was pyrolysed at 450 ℃ for 2 h under oxygen-limited conditions after ramping at 10 ℃ min^-1^. The resulting material was cooled, ground and passed through a 200-mesh sieve.

FeBC was prepared by impregnating corn-straw powder with 1 mol L^-1^ FeCl_3_ solution at a solid:liquid ratio of 1:100, stirring for 24 h in the dark, filtering and washing until no obvious yellow colour remained, drying at 60 ℃ and pyrolysing under the same thermal conditions as BC.

CaBC was prepared by mixing corn-straw powder with anhydrous CaCl_2_ at a mass ratio of 1:1 in water, stirring for 24 h, filtering, washing until chloride was no longer detected by AgNO_3_, drying at 60 ℃ and pyrolysing under the same thermal conditions.

SeBC was prepared by reacting BC with 0.05 mM Na_2_SeO_3_ solution (1:200, w/v) at room temperature (25 ℃) in the dark for 12 h. Upon completion of the reaction, the mixture was filtered, and the filter cake was repeatedly washed until the conductivity of the eluate approached that of pure water. The resulting solid product was dried to constant weight at 60 ℃, then ground and sieved through a 200-mesh screen.

**S2. Biochar characterization**

Surface morphology and elemental composition were examined using a field-emission scanning electron microscope (Zeiss Sigma 300) coupled with an energy-dispersive X-ray spectrometer (Oxford Xplore 30). Samples were sputter-coated with gold before observation, SEM imaging was performed at 5-15 kV, and EDS was used for semi-quantitative analysis of major surface elements. Specific surface area and pore characteristics were determined using a Micromeritics TriStar 3020 based on N_2_ adsorption-desorption at 77 K after vacuum degassing for at least 6 h. Specific surface area was calculated using the multipoint BET method, mesopore-size distribution using the BJH model based on the desorption branch, and micropore characteristics using the t-plot method. XPS analysis was performed using a Thermo Scientific K-Alpha spectrometer with Al Kα radiation (1486.68 eV); survey and high-resolution spectra were collected at pass energies of 160 and 50 eV, respectively, and binding energies were calibrated to the C1s peak at 284.80 eV. Peak fitting and chemical-state assignment were conducted using Avantage software. Raman spectra were obtained using a Renishaw inVia Reflex system with a 532 nm laser over 800-2000 CM^-1^, and the ID/IG ratio derived from the D (~1350 CM^-1^) and G (~1580 CM^-1^) bands was used to evaluate carbon structural order and defects. FTIR spectra were recorded using a Thermo Nicolet iS5 with the KBr pellet method over 4000-400 CM^-1^ at 4 CM^-1^ resolution with 32 scans, and major absorption bands were assigned according to standard infrared spectral databases.

**S3. N_2_O flux measurement and cumulative-emission calculation**

Before each gas measurement, jars were opened for 20 min. An initial headspace sample was collected, the jar was closed for 6 h, and a second sample was then collected. Pressure was balanced during sampling through the two-channel sampling assembly.Because two concentrations were measured per closure, the rate term was ΔC/Δt=(C₆ h − C₀ h)/6 h; a multi-point linear-regression criterion was not applicable. The flux was calculated as:

$$\text{F}\text{＝}\text{ρ×}\frac{\text{V}}{\text{m}}\text{×}\frac{\text{△C}}{\text{△t}}\text{×}\frac{\text{273}}{\text{T}}\text{×}\frac{\text{28}}{\text{42}}$$

Where: F is the gas emission flux (μg N kg^-1^ dry soil h^-1^); ρ is the density of N_2_O under standard conditions (1.978 kg m^-3^); V is the headspace volume of the culture bottle (L); m is the dry weight of soil sample (g); dC/dt is the change rate of gas concentration in a specific closed time period; T is the absolute temperature (K); The factor 28/44 converts N_2_O mass to N_2_O-N.

**Supplementary Tables**

**Table S1. Selected physicochemical properties of the loess and black soils used in the incubation experiment.**

| Property | Loess soil | Black soil |
| --- | --- | --- |
| Total N (%) | 0.11 | 0.922 |
| Organic matter (g/kg) | 19.1 | 38 |
| Available P (mg/kg) | 53.6 | 20.6 |
| Available K (mg/kg) | 116 | 294 |
| CEC (cmol(+) /kg) | 12.4 | 56.8 |
| 0.2-2 mm fraction (%) | 0.9 | 47.78 |
| 0.02-0.2 mm fraction (%) | 45.2 | 37.82 |
| 0.002-0.02 mm fraction (%) | 37 | 9.6 |
| <0.002 mm fraction (%) | 13.2 | 4.8 |
| Texture class | Loam | Sandy |

**Supplementary Figures**

**Figure S1. Surface morphology and elemental composition of unmodified and modified biochars. (a-d) SEM images and (e-h) EDS spectra of BC, FeBC, CaBC and SeBC, respectively.**

SEM and EDS showed that BC retained a porous honeycomb-like structure. FeBC contained Fe-rich surface deposits, with Fe accounting for approximately 8.2 wt% in the analysed surface region. CaBC showed dense layered or flocculent Ca-bearing surface features, with Ca accounting for approximately 5.6 wt%. SeBC retained an open porous surface with dispersed Se signals, with Se accounting for approximately 1.3 wt%.

**Figure S2. Pore structure of unmodified and modified biochars. (a-d) N_2_ adsorption-desorption isotherms and (e-h) BJH pore-size distributions of BC, FeBC, CaBC and SeBC, respectively.**

N_2_ adsorption-desorption curves showed type-IV behaviour for the biochars, supporting a mesoporous structure. FeBC showed a bimodal pore-size distribution, CaBC had larger pore features, and SeBC had lower pore volume and fewer small pores than BC. These features are relevant to microsite regulation because pore architecture can influence substrate retention, gas diffusion and micro-scale redox heterogeneity.

**Figure S3. Principal coordinate analysis of bacterial community structure based on Bray-Curtis dissimilarity.**

“all” includes every sequenced sample retained after quality control; “core” is the predefined 25 ℃, 2% CK/BC/FeBC/CaBC/SeBC comparison. Point colour denotes treatment, point shape denotes soil, and ellipses, where shown, are 95% group confidence ellipses.

**Figure S4. Non-metric multidimensional scaling of bacterial community structure based on Bray-Curtis dissimilarity.**

“core” is the 25 ℃, 2% CK/BC/FeBC/CaBC/SeBC comparison; and “all_D7” is the day-7 subset. Point colour denotes treatment, point shape denotes soil, and the reported stress quantifies ordination fit.

**Figure S5. Relative abundance and standardized treatment responses of extended candidate bacterial genera in loess soil.**

**Figure S6. Spearman correlation matrix among microbial, functional-gene, soil-chemical and N_2_O variables.**

Tile colour denotes Spearman's r. * and ** denote Benjamini-Hochberg-adjusted P < 0.05 and P < 0.01, respectively; unmarked cells did not meet the adjusted threshold.

**Figure S7. Gene-microbe-environment association network based on significant Spearman correlations among functional genes, bacterial taxa, soil-chemical variables and cumulative N_2_O emissions.**

Nodes were limited a priori to nirK, nirS, nosZ, the nosZ/(nirK + nirS) potential source-sink index, selected responsive genera, pH, NH_4_^+^-N,NO_3_^-^-N, and cumulative N_2_O-N. Edges are Spearman associations satisfying |r| > 0.6 and Benjamini-Hochberg-adjusted P < 0.05. Node colour denotes variable type, edge colour denotes association sign, and edge width is proportional to |r|.
